# Competency-based education and training for Community Health Workers: a scoping review

**DOI:** 10.1186/s12913-025-12217-7

**Published:** 2025-02-17

**Authors:** Marium A. Sultan, Emily Miller, Roosa Sofia Tikkanen, Shalini Singh, Arpana Kullu, Giorgio Cometto, Siobhan Fitzpatrick, Onyema Ajuebor, Nicholas Gillon, Anbrasi Edward, Youri P. Moleman, Shivani Pandya, Inyeong Park, Jung Yu Shen, Yefei Yu, Henry Perry, Kerry Scott, Svea Closser

**Affiliations:** 1 Johns Hopkins Bloomberg School of Public Health, Baltimore, MD USA; 2 Center for Global Health Inequalities Research, Institute for Sociology and Political Science, Faculty of Social and Educational Sciences, Norwegian University of Science and Technology, Trondheim, Norway; 3 Johns Hopkins India Private Limited, New Delhi, India; 4 School of Health Systems Studies, Tata Institute of Social Sciences, Mumbai, Maharashtra India; 5 Health Workforce Department, World Health Organization, Geneva, Switzerland; 6 Johns Hopkins University School of Education, Baltimore, MD USA; 7 KOFIH (Korea Foundation for International Healthcare), Seoul, Republic of Korea; 8 College of Education, University of Washington, Seattle, WA USA

**Keywords:** Community Health Workers, Education, Training, Competency-based Education, Scoping Review, Health Workforce

## Abstract

**Background:**

Community Health Workers (CHWs) play a critical role in supporting the delivery of health services globally. Competency-based learning programs can improve the transfer of learning to practice. This scoping review aims to characterize the published literature on competency-based education as an instructional and curricular strategy in community health worker training programs. We conducted a scoping review of the literature to identify how, by who, and in what ways CHWs are trained using competency-based education; and to characterize the extent of available evidence, as well as the gaps in that evidence.

**Methods:**

We conducted a review of the peer-reviewed literature on CHW competency-based education and training published between January 2010 to March 2023, drawing from four databases: EMBASE, OVID Medline, Web of Science, and CINAHL. We followed the PRISMA guidelines for scoping reviews. A total of 713 articles were reviewed and 236 were included for extraction based on the inclusion and exclusion criteria. Due to methodological heterogeneity, results were analyzed and synthesized only through a descriptive approach.

**Results:**

The literature on competency-based CHW education and training is most voluminous in high income contexts, primarily the USA. Overall, the included studies described very small-scale training interventions. Study types included observational (qualitative, quantitative, mixed methods, case studies) intervention or experimental studies, systematic or scoping reviews, and literature reviews. The most common practice area included was ‘promotive and preventive services’, whereas ‘personal safety’ was the rarest.

Learning programs tailored to CHWs with low-literacy, content tailored to local cultural contexts, and curricula that were co-designed with CHWs were identified in the literature as effective strategies for converting learning to practice.

Information on institutional support for CHWs was not provided in most of the articles reviewed. While the focus of our review was on education and training and not broader supports for CHWs, we still found it notable that training was usually discussed in isolation from other related supportive factors, including professionalization and career progression.

**Conclusions:**

We found considerable academic interest in utilizing competency-based education to support CHWs and improve their work, yet this exploration was largely limited to smaller, ad hoc programs, in high income settings.

Learning programs should be tailored to the realities and practice requirements of CHWs. Further work should illuminate the extent to which the design and delivery of education and training activities lead to acquiring and maintaining the requisite competencies.

**Supplementary Information:**

The online version contains supplementary material available at 10.1186/s12913-025-12217-7.

## Introduction

Considering the anticipated shortage of about 10 million health workers by 2030 [1], and the urgency to achieve the goals for Universal Health Coverage and Sustainable Developmental Goals, countries are increasingly prioritizing Primary Health Care-oriented approaches to health service delivery. This has direct implications on the health workforce. Universal Health Coverage relies on a health workforce that is responsive to the communities they serve.

Community Health Workers (CHWs), when embedded in and adequately supported by primary care teams, can play a critical role in supporting the delivery of health services globally. Evidence from published literature has established CHW effectiveness in improving maternal and child health outcomes [2, 3], as well as controlling non-communicable [4, 5] and infectious diseases [3, 6]. Countries like Brazil, Ethiopia, India and Nepal have shown demonstrated success across the care continuum [7–9].

The diversity of health system needs and contexts has resulted in a broad variety of CHW roles and qualifications. There is wide variation in training duration as well – from two years for a *behvarz* in Iran to 5 days for a Community Health Volunteer in Ghana [10]. Previous reviews have noted a lack of information on best practices for CHW education due to the difficulty of obtaining education content, compounded by the complexity of synthesizing varying education approaches and designs across diverse contexts [11–13].

While traditional educational approaches focus largely on knowledge retention, competency-based education centers on continuous, real-world application of learning, such as problem solving and decision making [14]. Competency-based education has been recommended as an effective approach to preparing health workers to respond to evolving scopes of practice and deliver patient-centered care [15], and has been observed to improve clinical performance [16].

When competency-based education programs are designed together with communities, education can prepare CHWs to better meet the needs of the populations they serve, through adapting to emerging health challenges, tailoring their approach to address specific community needs, and responding dynamically to evolving health priorities, ensuring a more agile and responsive healthcare workforce [17].

In recognition of the reported challenges and paucity of evidence and recommendations for CHWs, in 2018 the World Health Organization (WHO) published guidance on health policy and system support for CHW programs, including on competency-based pre-service CHW education that would help countries formalize CHWs into the health system architecture and help sustain their impact. However, many countries are yet to integrate these recommendations, and continue to face broader challenges related to supervision, retention, low motivation, and funding. These critical factors must also be considered in the design of education programs that are tailored to what is feasible for CHWs to do [12, 18–21].

With the renewed recognition and prioritization of CHW programs following the adoption of a dedicated resolution at the 72nd World Health Assembly in 2019 [22], countries have begun to accelerate their efforts to expand the number of CHWs in the health workforce. This will require investments in contextually relevant pre- and in-service education and training with relevant, reliable, and consistent support measures.

Therefore, there is a need to move towards competency-based education (CBE) and lifelong learning [17]. Competency-based education improves the application of learning to practice and helps learners perform within their own health system contexts [23]. To inform the development of a CHW competency-based prototype curricula guide, we conducted a scoping review of the existing body of scientific literature on competency-based education for CHW programs. This review focused on how CHWs are trained using competency-based education, and where the evidence is lacking. This aim of this scoping review was to characterize the published literature on competency-based education for CHWs and draw out instructional and curricular strategies from these learning programs.

We aimed to characterize *who* was providing the training in the literature, *what* was covered in the training, and *how* trainings were delivered. We also aimed to characterize the evidence on connections between competency-based trainings and broader supports for CHWs, such as career progression. We cast a wide net, aiming to collect all published articles on CHW competency-based training that contained details about what was being taught, by whom, and how.

## Methods

Our rapid scoping review was guided by the steps outlined by Arksey & O’Malley and the Joanna Briggs Institute for scoping reviews [24, 25]. We made modifications to this methodology to facilitate timely completion of the review, in accordance with rapid review methodology suggested by the Cochrane Group and others [26, 27] for reviews conducted within a time frame of less than a year. We report our findings using the Preferred Reporting Items for Systematic Reviews and Meta-Analyses Extension for Scoping Reviews (PRISMA-ScrR) by Tricco et al. [28] (Annex 1).

First, we implemented a search strategy on CHW competency-based training. Subsequently, teams of researchers developed the inclusion and exclusion criteria, conducted the title and abstract screening, and conducted the full-text screen and extracted data onto the data charting form. Once data was charted, groups of researchers processed the charted data for analysis. For the purpose of the search strategy, we considered the terms ‘education’, ‘training’, ‘education and training’, and ‘learning programs’ as closely related to indicate teaching of any kind directed towards CHWs, given the variable meaning associated to these terms in the underlying literature.

### Search strategy

We implemented a search strategy to identify articles on CHW competency-based training across four databases: EMBASE, OVID Medline, Web of Science, and CINAHL. The search was conducted to identify papers published from January 2010 to March 2023 to capture the most recent practice in CHW training from preprint and published articles with empirical designs (observational or experimental) and review articles.

The search strategy was developed in collaboration with research librarians working at Johns Hopkins Bloomberg School of Public Health’s Welch Library. CHW search terms were derived from The International Labor Organization definitions of a CHW in the International Standard Classification of Occupations (ISCO) 2008 code ISCO-08 3253, and cadre names mentioned in existing systematic reviews on CHWs [13, 29–33], as well as a CHW systematic review Protocol [34]. In addition to 100 + terms aimed at identifying CHW related papers, terms were added to narrow our scope to education or training. These educational terms were created mainly through discussions with research librarians about our goals, and following subject headers and categories (e.g., MeSH) trees to identify all related words for each individual database. Our search strategy is detailed in Annex 2.

### Article screening and data collection

The title and abstract screening was conducted using Rayyan. A small sample of abstracts were reviewed individually and from conversations about these decisions, a rigorous inclusion and exclusion rubric was created and used for the rest of the screen (Annex 3). Included articles were of empirical design, in the English language, and focused on the training of Community Health Workers that was competency-based.

Each abstract was screened by two reviewers. Each reviewer made their decisions independently, without seeing the decision of the other reviewer. During weekly group calls the ‘blind’ mode was turned off to compare reviewer decisions and reconcile any conflicts.

In the full-text screen an additional criterion was added, stating that in order to be included, an article must describe a competency-based training. To meet our definition of competency-based training, the article had to include at least two of the following dimensions: knowledge, skills, attitudes, behaviors. These dimensions were drawn from the definition of competency-based education (CBE) that was proposed by WHO as ‘an outcomes-based approach to curricular design, development and implementation that emphasizes the mastery of learning, and the application of knowledge, skills and attitudes in the context of performance, rather than the process of learning and the acquisition of knowledge, skills and attitudes [35]. The fourth dimension was used to identify articles that emphasized applied learning, as this is a specific requirement of CBE [35].

A data charting form for published literature was developed in Microsoft Excel. Data extracted from the articles selected for full-text review included contextual study information (i.e., authors, year of publication, study type); the training curriculum and assessment described; information about the CHWs; curricula contents; and structural support around the training (Annex 4).

The curricular domains used to categorize training content were informed by the domains stipulated in WHO's guideline on health policy and system support to optimize community health worker programmes [1]. However, after review of existing competency frameworks it was decided that some curricular domains could be stratified into smaller but relevant domains. Before extracting data from the literature, all the 12 curricular domains were defined in terms of the CHW tasks and relevance for training in this practice area. The definitions of CHW tasks for each domain were utilized by data extractors to identify domains from the content described in the training curriculum description, using their best judgement, regardless of whether the articles mentioned the domain by name. As these domains are not discrete and were often integrated in practice, we identified domains based on the training curriculum itself, as described in the article. Multiple domains could be identified from a single article. For example, if training on a specific preventive health service covered the social determinants of health, we would identify both “promotive and preventive services” and “social and environmental determinants” as domains.

### Synthesis methods

Once the extraction was completed for all records, data was processed for analysis. Processing included standardizing inputs, transforming multifaceted columns into binary columns, and creating binned categories of certain variables.

Data was quantitatively analyzed to determine counts of key variables, and to provide descriptive statistics describing the distribution of those variables. Sometimes we stratified the descriptive statistics to provide additional information (for example, to compare training modalities described in high income countries to those described in low and middle income countries). Processing and analysis were done using Microsoft Excel, Python on jupyterlab, and STATA.

For the key findings column, which generally consists of free text from the findings or conclusion of the article or article abstract, this group of reviewers created a codebook using both inductive and deductive methods. Each reviewer coded 5 articles for key findings, and the group came back together to discuss and iterate the codebook. Once the codes were decided on, the entire dataset was divided for coding, which was done directly in Microsoft Word, with results pulled into an Excel sheet.

Critical appraisal (‘risk of bias’ assessment) of individual studies was not conducted, given the heterogeneity in the study designs and the fact that this is an optional step in scoping reviews [36]. Similarly, an assessment of risk of bias across the body of evidence (all included studies) was not performed, given that this is not congruent with the aims of a scoping review [36].

## Results

### Selected articles

**Fig. 1:**
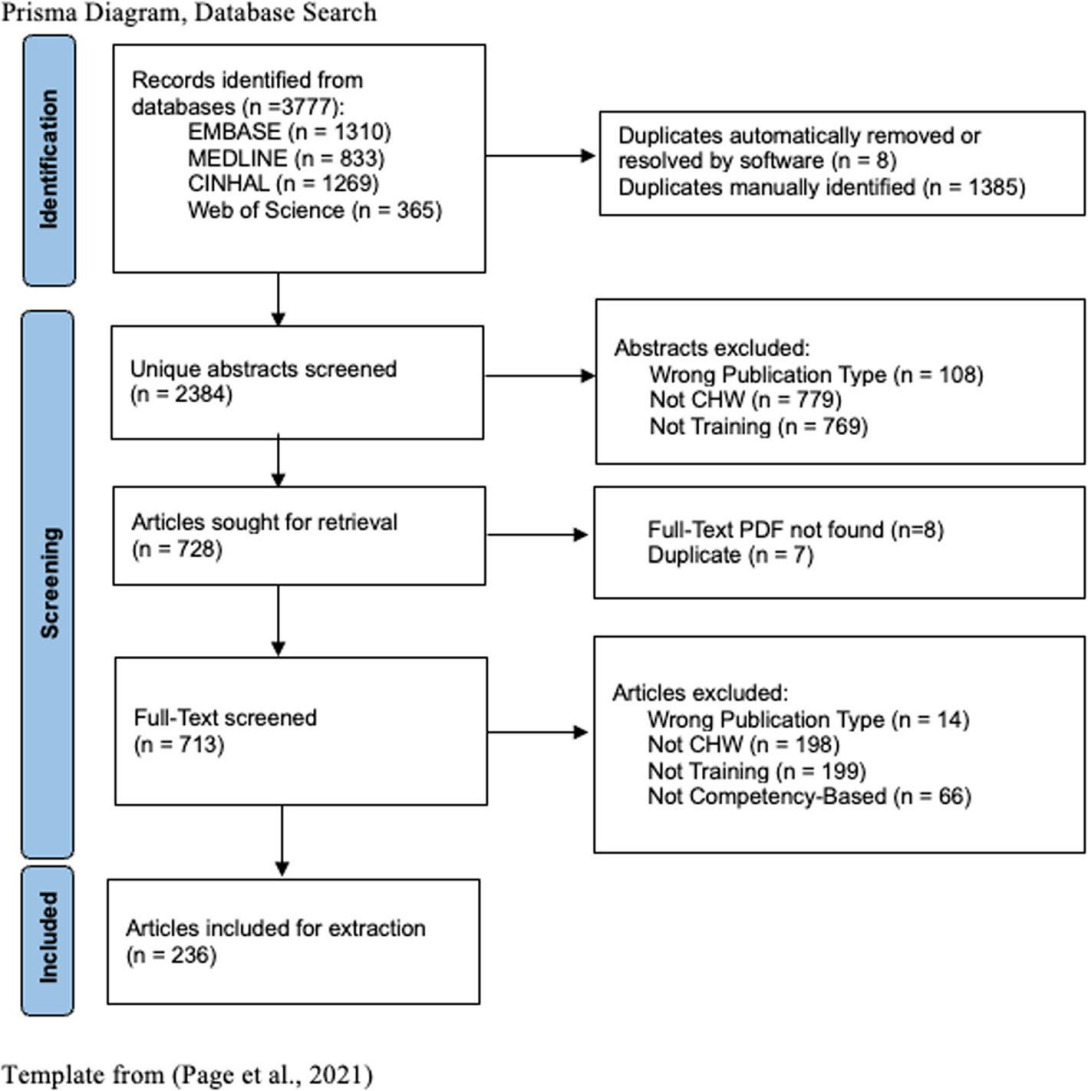
PRISMA diagram of database search

Three thousand seven hundred seventy seven records were identified in the comprehensive search conducted in March 2023. 1393 were determined to be duplicates, and 2384 unique abstracts were screened based on our exclusion criteria (Annex 3). After abstract screening, 728 articles remained. These were sought for retrieval and 713 unique full-text articles were able to be found. After full-text screening, 236 articles were selected for data charting and analysis (Fig. 1) [37].

### Features of included articles

#### Included articles by year

**Fig. 2:**
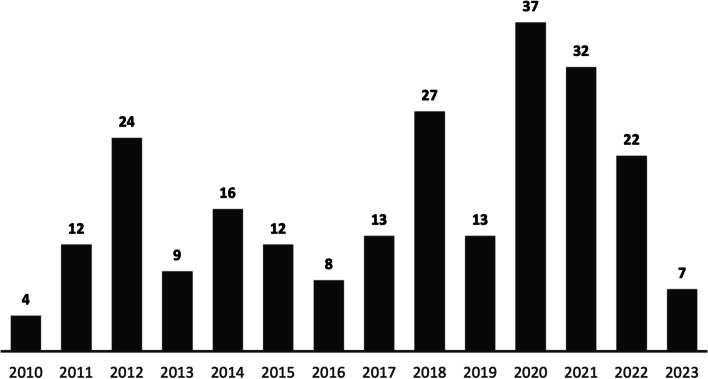
Count of included articles by year of publication

There has been an increase in literature that met our inclusion criteria on competency-based CHW trainings over time, with the largest spike in 2020. In general, more studies were included from recent years (Fig. 2). The lower volume of search results from 2023 reflected the search being conducted in March of that year.

#### Study design

The most common study design in the included articles was intervention studies (*n* = 115). Almost as common (*n* = 102) were observational studies, where a training was conducted and described, but there was no explicit comparison group. Reviews (*n* = 13) and methodological reports (*n* = 6) were also extracted.

#### Regions and income levels

**Fig. 3:**
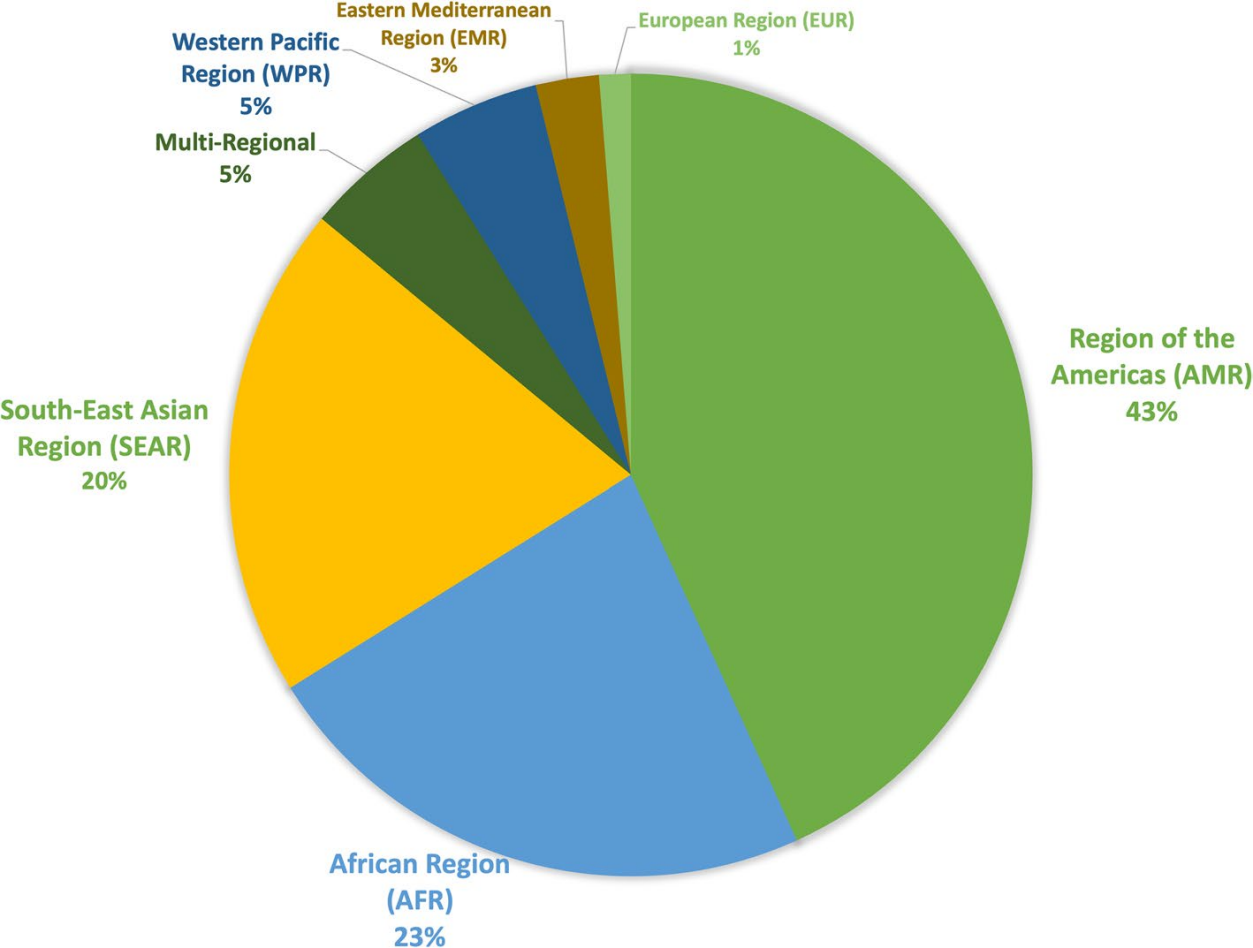
Count of included articles by WHO region

The included articles, as shown in Fig. 3, were overwhelmingly focused on the American Region (AMR, *n* = 102), which occurred in the literature at almost twice the number of the second most occurring region (AFR, *n* = 54). The region with the least results was Europe, with only 3 included studies.

Articles from higher-income countries were more common in our search compared to articles from lower-income countries (Table 1). Income and region were highly correlated with countries in the Americas (85/102) and Europe (2/3) tending to be high income, SEAR (39/47) mostly LMICs, and AFR mainly lower income (31/54).

**Table 1:** Count of articles by World Bank country income level (2021 designation)

Income Designation	Count
High Income (HIC)	92
Lower-Middle Income (LMIC)	64
Upper-Middle Income (UMIC)	37
Lower Income (LIC)	31
Multi-Country (MC)	12
**Total**	**236**

The most common lower-income country in the literature was Malawi (*n* = 13). India was the most common lower-middle income country (*n* = 25), and South Africa the most common upper-middle income country (*n* = 14). The USA (high-income, *n* = 84) was the most common country in the literature, across income levels.

### Who trains CHWs?

#### CHW trainers

The studies reviewed used three main approaches as to who would carry out CHW education and training. One approach was to have training sessions conducted and facilitated by subject matter training experts, trained mentors, and tutors [38, 39]. These training topics were often focused on very specific content and were frequently one-off. A second approach was for trained peers to conduct the trainings [40, 41]. These CHWs sometimes possessed prior experience in training or had undergone specialized training to instruct fellow CHWs [42]. This “train the trainer” approach allowed standardized capacity strengthening in an efficient way. A third approach was self-learning, in which CHWs embarked on self-guided learning journeys by engaging with available training resources [43, 44].

#### CHW Program management type

A significant portion of articles did not specifically include information about the entity conducting the CHW training and therefore that information could not be extracted (*n* = 77). The entities conducting the training intervention described in the remaining articles were governments or government collaborations (*n* = 69), followed by non-governmental organizations (NGOs) (*n* = 49), with a lesser number of academic institutions (*n* = 19). A total of 22 articles reported the collaboration of two or more institutions in conducting training interventions.

#### Scope

Most articles included in our analysis describe a single, ad hoc training intervention, and in these instances, we captured how many trainees were included in the training intervention. Overall, our studies described very small-scale training interventions. The outlier was a large-scale study that included 45,700 Lady Health Workers (LHWs) throughout Pakistan [45], almost half of the LHWs in the country at the time. The smallest training program described was in Senegal [46] with just one trainee. Of the articles where the number of trainees trained was readily available, the average number of trainees was 393, falling to 132 without the outlier of the one LHW study. Not all articles provided a specific count of the trainees discussed, as they focused on detailing the training in existing CHW programs rather than emphasizing a specific training intervention [47].

### What are CHWs trained in?

#### Training curriculum

Given the diversity of roles and responsibilities CHWs are expected to have in practice, it can be challenging to compare various training content [48]. In 2018, based on a critical appraisal of globally available evidence, WHO suggested a set of six “core” and one “additional” curricular domains for pre-service training of CHWs, assuming that these are relevant to the expected roles within their contexts [49].

**Fig. 4:**
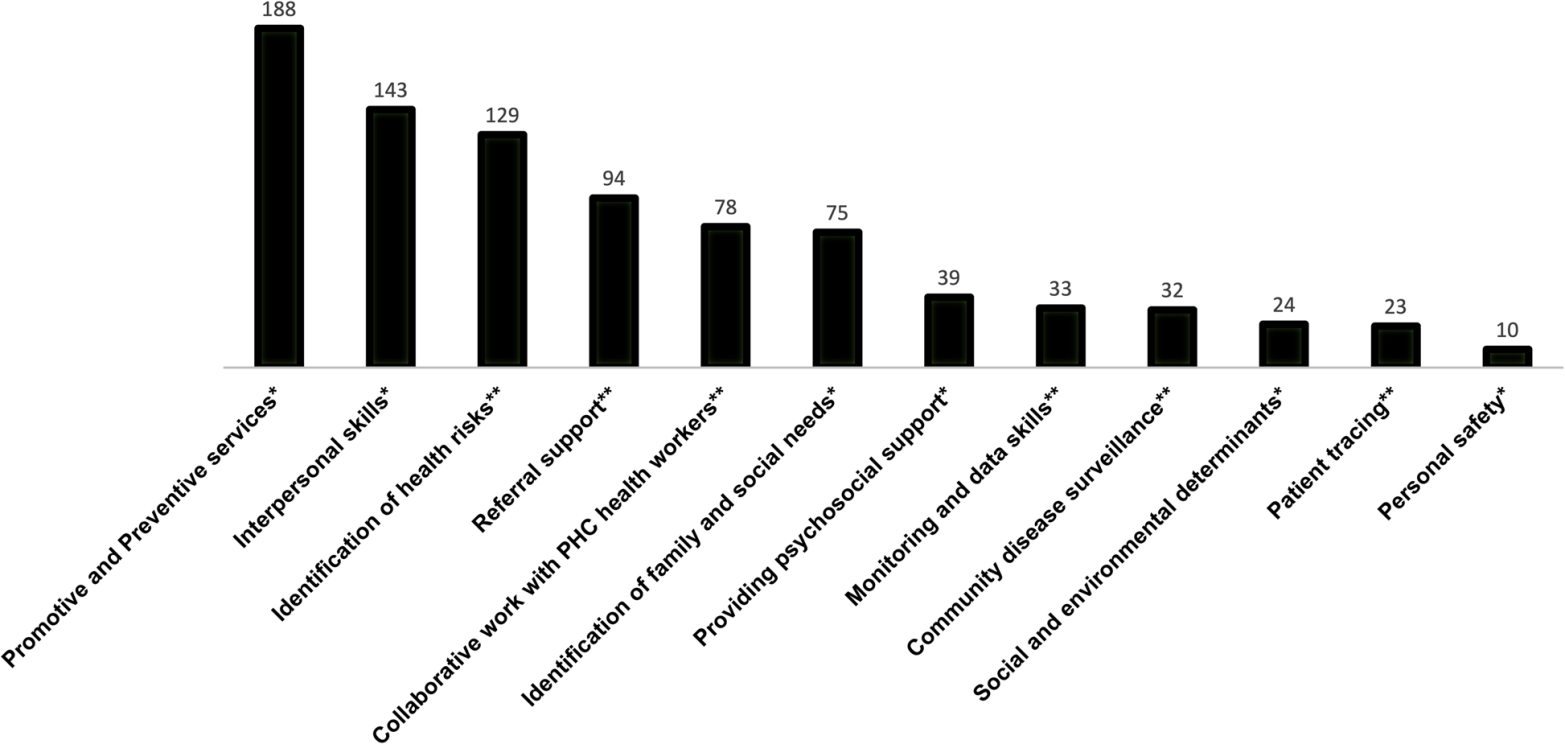
Count of WHO Curricular Domains (one article may have multiple domains mentioned). *indicates domain as indicated in 2018 WHO CHW Guideline [49]. **indicates domain subdivided from larger domains

The prevalence of specific curricula content in the reviewed articles was mapped to these seven domains (Fig. 4), using details of the training from the article, ideally the curriculum itself. Four domains were used as indicated, namely personal safety; interpersonal skills related to confidentiality, communication, community engagement and mobilization; providing psychosocial support; and social and environmental determinants of health. Two of the core domains included multiple distinct components, each big enough to comprise a domain of their own. The domain called “promotive and preventive services, identification of family health and social needs and risk” was subdivided into its three component parts: 1) promotive and preventive services, 2) identification of family and social needs, and 3) identification of health risks. The domain called “integration within the wider health care system in relation to the range of tasks to be performed in accordance with CHW role, including referral, collaborative relation with other health workers in primary care teams, patient tracing, community disease surveillance, monitoring, and data collection, analysis and use” was divided into its five component parts: 1) referral support; 2) collaborative work with other health workers; 3) patient tracing; 4) community disease surveillance; and 5) monitoring data, collection, and use. If a training described in an article covered multiple domains, it was coded with all those domains.

#### Relevance to the CHW curricular domains

The most common domain included in CHW trainings from our literature review was ‘promotive and preventive services’, followed by ‘interpersonal skills’ and ‘identification of health risks’. ‘Personal safety’ was the rarest training domain, indicating a gap in the literature on training for personal safety.

In addition to the above domains, we also found studies documenting trainings focused on equipping CHWs with research skills including community based participatory research [50] and research fundamentals [51, 52] as tools to improve factors like quality of care or job performance. One training program found that providing training in research fundamentals facilitated the ability of researchers to better communicate with CHWs and CHWs with their communities; similarly, another training program that integrated research fundamentals underscored the importance of continuous mentoring and coaching for CHWs [53, 54].

#### CHW Curricular domains by income and region

Across nearly all incomes and regions (except Europe), ‘promotive and preventive services’ was the most common curricular domain covered in the trainings reviewed. This reflects the centrality of this role for CHWs across the globe.

There were differences between the frequencies of domains found in articles describing trainings from HICs versus LMICs. ‘Personal safety’ was found in a higher proportion of articles in HICs (8/92) than in articles in LMICs (1/64) or LICs (1/31). The ‘social and environmental determinants of health’ domain was not identified in any articles describing trainings from low-income countries, but was sometimes included in articles describing training in HICs (16/92) and UMICs (4/37). In the literature, the trainings described in LMICs tended to to focus less on the larger structural and systemic issues that contribute to health outcomes, focusing instead on immediate skills and practices. ‘Interpersonal skills’ featured in 50% or more of articles describing trainings across income levels, but were most common in articles describing training in HICs (65/92, 71%). Curricular content related to this domain was included in all 3 articles about trainings based in Europe.

Often, curricular content related to ‘personal safety’ and ‘social and environmental determinants of health’ featured in articles with no reference to a specified health service.

#### Health services

**Fig. 5:**
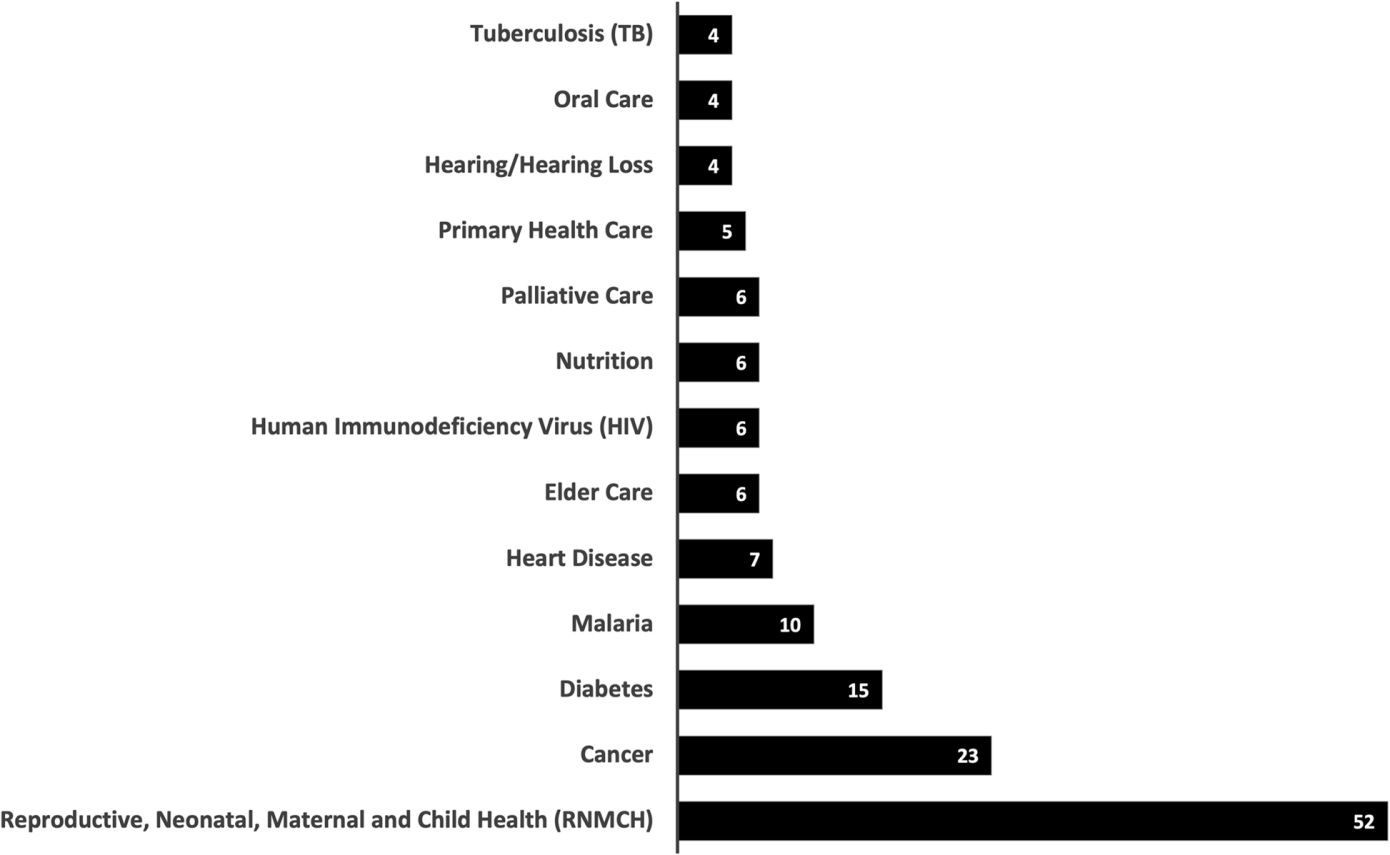
Count of top 15 Health Services Extracted (one article may include multiple health services)

We also categorized the articles by the broad topics of the trainings they provided to CHWs (Fig. 5). The most frequently cited content specific to a health service was Reproductive, Neonatal, Maternal and Child Health (RNMCH) – although this accounted for less than 25% of all articles. For 44 studies, a specific health service could not be extracted.

We found that education and training programs described in these articles often linked multiple health services. There were no strong trends in which particular combinations of health services were trained together in these articles. However, more common groupings included training asthma, heart disease, or nutrition along with diabetes; RNMCH along with Water, Sanitation and Hygiene (WASH); and palliative care along with rehabilitation.

#### Health services trained, by income and region

Health services covered in the trainings described in these articles varied by both country income and region. The most common health services covered by CHW trainings in the articles in high income countries were cancer (19/92), diabetes (8/92), and mental health (7/92). Cancer and diabetes were not identified in any trainings based in lower income countries in the articles we reviewed, but mental health services appeared in similar proportions as in HIC contexts (3/31).

In lower (10/31) and lower-middle income (24/64) countries, the vast majority of trainings in the articles focused on Reproductive, Neonatal, Maternal and Child Health (RNMCH).

Regionally, South-East Asian countries had the largest proportion of trainings focusing on RNMCH in our review (18/27), followed by Africa (20/53), while in the Americas the proportion of trainings focused on RMNCH was much lower (10/102). In Africa the second most common health service covered in the articles, after RMNCH, was malaria (5/53). In South-East Asia it was mental health (5/27).

Our extraction did not capture any trainings specifically for neglected tropical diseases, another large gap in the literature reviewed.

### How are CHWs trained?

We captured considerable variability in teaching and learning methods described in the articles about CHW trainings across different contexts. Although training methods varied widely, there was a common thread in the articles to contextualize trainings to important factors such as CHW background.

#### Training length

**Fig. 6:**
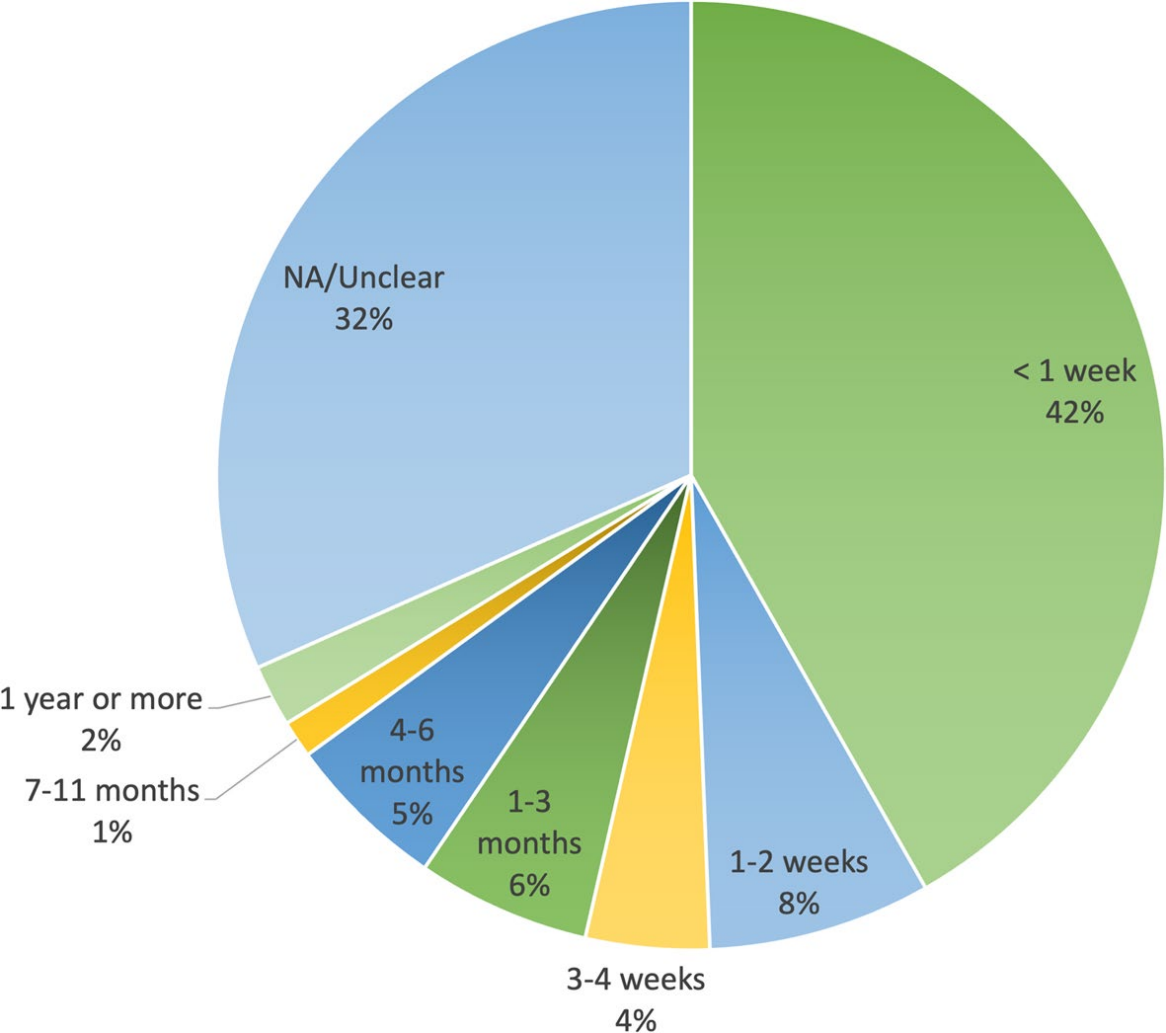
Training Duration (*n* = 236)

The length of the education and training programs in our sample varied greatly (Fig. 6). Differences in the way the information was reported in the articles made it difficult to compare studies directly. However, some general trends were apparent. Almost half of education and training programs (41.8%) described in the articles were a week long or less, and 12% were between 2–4 weeks long. 11.4% of trainings ranged from 1–6 months, but it should be noted that many trainings in this category were not full time, consisting of intermittent contact with CHWs. Very few exceeded one year (2.1%).

#### Training modalities

In-person trainings are commonplace in the literature in addition to other modalities. Most articles in our review referred to CHW education and training delivered via in-person methods [50, 55] using teaching modalities such as seminars and workshops. For subjects necessitating the acquisition of clinical knowledge and skills, such as Integrated Management of Childhood Illnesses, skilled birth attendance, breastfeeding, and asthma management, the articles predominantly described traditional classroom-style instruction. These pedagogical approaches included lectures, practical demonstrations, skill practice sessions, group discussions, instructor feedback, and collaborative problem-solving among peers [51].

The literature also contained examples of using a “blended” approach, combining both online and in-person and/or self-paced offline components [52, 56] allowing in-person contact hours whilst also increasing flexibility and reducing the burden on CHWs to attend exclusively in-person trainings. Distance education was considered a feasible approach for some CHWs in geographically isolated regions [57, 58]. Online training was found to have value in increasing reach, but may not be feasible in all environments [59].

#### Pedagogical approaches

The focus of competency-based education on cultivating practical skills and knowledge acquisition relies on tailored instructional methods that emphasize mastery of specific competencies and educational strategies that cultivate abilities for real-world application. Interactive, practical sessions mirroring real-world scenarios emerged as a prevailing strategy for skill reinforcement in the literature. In these articles, learners were encouraged to actively participate in discussions and contribute to peer-to-peer learning, thereby enhancing the application of learning. Additional pedagogical approaches to utilize and/or replicate real-world scenarios included experiential learning [60], home visits [61], and role play [62]. Additionally, practical tools such as checklists and pocket guides were found to be helpful in serving as memory triggers during service delivery [63, 64].

Articles described considerations for trainings to accommodate different learning styles, with programs incorporating elements to target a variety of learning approaches. An in-service training of *promotoras* on stress reduction targeting individual behavior change among Latina immigrants in the U.S. used active learning styles including visual, kinesthetics, affective, and cognitive styles [55]. A training for CHWs elder care in the Philippines provided a wide variety of training content and activities with intentional consideration for diverse learning styles [65].

Our review yielded multiple ways to tailor training methods for low-literacy CHWs [50, 59, 66–69]. One training program in Zambia, for example, adopted a simplified curriculum for neonatal resuscitation protocol and administration of oral antibiotics for traditional birth attendants with limited literacy and numeracy skills, improving their knowledge and skills [70]. Other articles for low-literacy CHWs employed pictorial, action-based checklists [66, 71], educational videos [67], and hands-on mannequin-based practice [68]. A number of articles discussed pedagogical approaches designed to tailor content to be relevant and effective to the context in which CHWs work and live. There were multiple articles focusing on including cultural sensitivity in trainings [50, 53, 72, 73] and culturally tailored curricula [51, 52, 74–77].

The pedagogical approach underlying many CHW training programs in our sample adhered to the principles of popular education [38, 76, 78–87]. Popular education is an epistemic approach that draws on the expertise of trainees and can empower individuals and communities [88]. These programs featured participatory methodologies and emphasized values related to equity and social justice.

#### Co-design and lived experience

Many articles noted the importance of including community health workers, community members, and other key stakeholders in the development of the training [55, 89–91]. Co-development was found to help ensure cultural relevance [91, 92], a factor noted as essential to program success [51, 52, 74–77]. A study which integrated the lived experiences of CHWs in a cancer education training program in Alaska, USA found that by centering the experience of communities in the training, CHWs improved their abilities to communicate about cancer risks, recommend cancer screenings and even improve their own health behaviors for cancer prevention [70].

#### Training assessment

Trainee assessments described in the articles were most often pre-post surveys to determine change in demonstrated knowledge and skill level before and after the education and training program. The Kirkpatrick Model of Evaluation [93], a training evaluation framework that emphasizes real-world applications of content learned, was used in some studies [43, 81, 94–97]. Of those that employed the Kirkpatrick Model, evaluations were largely focused on the immediate first and second levels, with little follow-up for longer term impact. Some studies included clinical assessments or shadowing of CHWs in the field post the education and training intervention, but this was relatively uncommon [98, 99].

Most studies measured the effect of training on increasing CHW knowledge, skills and/or attitudes and found a positive change. Studies that showed mixed results tended to show an increase in knowledge post-training but no change in skills [78, 100, 101]. Fewer studies looked at outcomes on clients, or the health system. The few that did focused on increased levels of care-seeking by community members from CHWs [102, 103].

### What are the institutional supports for CHWs?

#### Formalized credentials and licensing for CHWs

The majority of articles (*n* = 208, 87.8%) did not report information on if and how CHWs were licensed as a result of the training. Of those that did report this information, the majority provided a certificate to CHWs who had completed the training program [45, 63, 81–84, 92, 104–112]. Due to the wide variability of the education and training programs reviewed, there was heterogeneity in terms of issuing credential and licensing characteristics (e.g., a certificate of completion from an implementing partner versus formal recognition from an accrediting institution of the health system). In some cases, CHWs were required to take an exam to qualify for certification [47, 60, 110, 113]. For example, in one training program in the U.S., CHWs had to take the Certified Asthma Educator (AC-E) exam to become nationally licensed [60]. In Bangladesh, CHWs received skilled birth attendant training and received recognition from the Bangladesh Nursing Council as skilled providers [51]. CHWs in training programs implemented in South Africa and Guatemala were provided ID cards upon completion of training [113, 114].

#### Remuneration for training

Similarly, the majority of articles (*n* = 198, 83.5%) did not mention whether CHWs were renumerated for training. 27 articles reported some form of payment to CHWs – either as stipend, allowance, or in the form of transportation reimbursements [44, 45, 47, 52, 55, 59, 74, 80, 87, 94, 97, 112, 115–128]. Most of these articles were about training in the U.S. (*n *= 16), where CHWs were paid between $20-$250 USD for completing trainings [52, 55, 59, 80, 87, 112, 116–118, 120, 126]. In Taiwan, CHWs were provided monetary certificates worth $200 USD for completing oral health-related training [119]; in Cambodia, CHWs received $5 USD for diabetes prevention training [124]; and in Grenada, CHWs received $150 per training sessions around cervical cancer and HPV [115]. Some CHWs in the studies reviewed, mostly in LMICs, received transportation reimbursement for attending trainings [39, 60, 129–131]. In Tanzania, HIV-focused CHWs received $20 USD for integrating maternal and child health-related training [123].

#### Career progression for CHWs

The majority of studies did not mention any kind of career progression pathway related to CHW training. Five articles [51, 60, 84, 113, 132], reported on the pathway and potential of career progression of CHWs – though exact pathways were not described in detail. In South Africa, emergency first aid responders (EFAR) could be promoted to an advanced EFAR position [113], and in the US, CHWs known as ‘birth sisters’ could be promoted, though there was no information on the details of that progression pathway [132]. Other types of career progression identified included CHWs becoming trainers [51], CHWs receiving job placement support [84], and CHWs becoming licensed though a national body [60].

#### Additional support for CHWs

As mentioned earlier, the majority of the articles reviewed described pre-service trainings. Yet multiple articles stressed the importance of continual support, such as refresher trainings [75, 98, 133], especially for health workers with limited formal education. Several articles also emphasized the need for ongoing mentoring and supportive supervision within and post-training also emerged as a facilitator for CHW success [74, 134, 135].

### Embeddedness and integration

Embeddedness—linking the education and training of CHWs to the needs of the communities that they serve—emerged as a theme in the findings or recommendations of 10 studies. Areas of practice for which making such linkages was common were mental health services [91], health promotion and prevention [136] and health education [80]. Ensuring that training was aligned with the existing CHW responsibilities as well as community priorities was considered relevant for sustaining training outcomes. Three studies highlighted the anticipated benefits of embedding CHW training into existing programs [71, 116, 119]. Benefits included enhanced community capacity [71], improved needs assessments [116], and improved access to care [119]. Another three studies mentioned the need of embedding training into existing programs [136–138]. Specifically, they emphasized the need for sustained program support with the goal of achieving improved service accessibility and uptake after completion of the training [136–138]. One article pointed out that program support can support the adoption of evidence-based practice, teamwork, and innovative roles [137]. Another illustrated that institutional support for CHWs doing this complicated work is integral to the success of the program [47].

There were very few studies that clearly described integrated training, defined as training on health promotion for various health services being supported under the same care package. Yet, there were some notable examples of trainings that did explicitly prioritize integration [38, 47, 102, 139–141], with many of these being NGO-led CHW programs in the USA.

### Certainty of the evidence

Given the heterogeneity of the education and training programs reviewed, the lack of robust evaluations in the articles, small sample sizes in terms of CHWs trained with a focus on small-scale training programs and training programs in high income settings in the articles reviewed, we characterize the certainty of the evidence in this review as very low in all domains. Given that most articles reviewed were describing training programs of several hundred or fewer CHWs, and it is likely that most CHWs trained globally are given such training as part of large-scale national programs, there is strong reporting bias in this literature.

A full list of the articles reviewed is included in the Supplemental Material (Annex 5).

## Discussion

### Limitations

This review was completed to inform the development of a competency-based curriculum guide for CHWs. Therefore, we extracted articles that described training content, but not those that exclusively evaluated a training without providing information on content.

We used the definition of competencies detailed in the World Health Organization Global Competency and Outcomes Framework for Universal Health Coverage [35] in order to identify articles on competency-based education and training, but we are aware there are many other definitions. Competency-based education is sometimes regarded as a catchphrase, with differing interpretations and applications across training programs; so not all programs that incorporate competency-based principles explicitly identify as such, while some self-described ‘competency-based’ programs may not align with the definition we applied in our review.

This analysis was done to gain a better understanding of the scope of published literature; it seems from this work that the published literature is likely not particularly representative of the true scope of CHW competency-based training. Our results can only highlight features of trainings that were reported in the literature.

We found considerable academic interest in utilizing competency-based education to support CHWs and improve their work, yet this exploration was largely limited to smaller, ad hoc contexts. While the literature predominantly describes trainings that took place within large-scale national programs, it is important to note that the education and training initiatives described often targeted only a subset of these CHWs, with limited descriptions of the comprehensive curriculum offered to the broader CHW population.

Our findings suggest that interest in competency-based education for CHWs is growing, although papers documenting such trainings over represent higher-income contexts. Furthermore, the majority of the trainings in our review were small-scale intervention designs, with limited evidence of incorporating competency-based education into formal CHW programming. The disproportionate number of articles describing high income contexts and articles describing trainings given to a relatively small number of CHWs, limits our ability to draw inferences on CHW training and practice globally. We had difficulty finding consistent key details across different articles, leading to heterogeneity in the completeness of various datapoints collected. We did our best to mitigate bias by employing rigorous study design and implementing standardized protocols, but the variability in information presented in the articles nonetheless led to inconsistent information for some key variables. For example, due to heterogeneity in how articles reported training content, curricula domains could only be extracted across articles by best judgement, and this could have biased our comparisons between various types of training programs. There may have been cases when a training, in practice, included domains and competencies that were not reflected in the article regarding the training. However, we extracted to the best of our understanding based on the described training content contained in the article. Furthermore, publication bias may have resulted in underreporting in the literature of some of the features that are actually implemented by training programs.

### Interpretation

Few scoping or systematic reviews have explored CHW competency-based education and the learning outcomes of those education programs. One systematic review of existing reviews reported that training impacted CHWs’ motivation, job satisfaction, and community confidence, but that training duration and frequency did not show a consistent effect on CHW efficacy [32]. The authors recommended a knowledge and skills-based training approach to improve CHW self-efficacy and skill levels, with supportive supervision and mentoring. Another systematic review of CHW training programs for cardiovascular disease management in low- and middle-income countries reported that pre-post knowledge tests were inadequate to ensure practical application of knowledge gained, recommending validated mechanisms for evaluating CHW training programs [142]. A scoping review of CHW programs emphasized the importance of appreciating CHW perspectives and experiences following training to determine impact of the training program, as the traditional evaluation metrics of numbers trained, hours of training, and scores achieved were inadequate to assess effectiveness [11].

In the articles identified in our review, trainings for CHWs largely follow traditional North–South burden of disease paradigms that emphasize infectious and “tropical” diseases and maternal child health in poorer settings [143] and non-communicable diseases in high-income contexts. This is likely in part because CHWs in low- and middle-income countries have an appropriately greater focus on infectious disease and maternal-child health given the burden of disease in their settings. However, given the increasing burden of NCDs in many low- and middle-income countries, there is a need for more evidence on competency-based trainings in NCDs tailored to CHW work in low- and middle-income contexts.

There was no consistency in our findings with respect to factors like training duration or curricular fundamentals. This is understandable given the diversity of CHW roles, yet a basic set of universal competencies that could be adapted across settings might support regularization of standards to establish and strengthen quality of care globally. This analysis could further aid in developing a prototype curricula guide that includes universal, as well as context- and role-specific, competencies and practice activities for CHWs globally.

Crucially, while the way CHWs are trained matters, focusing narrowly on curricula alone is insufficient for transformative change. There was little information in the articles in this review on whether CHWs were formally recognized, credentialed, or provided career development opportunities after completing competency-based training programs. This suggests that there is scope for research exploring the relationship between competency-based trainings and broader supports. This is an especially important area for further research given that many CHW programs continue to be made up of women with heavy work responsibilities and low remuneration. The literature also focused predominantly on pre-service training with limited evidence for refresher trainings or on-the-job supervision, despite the fact that these are recognized as important support mechanisms for community health workers [21, 144]. There is therefore a need for additional research on continuing education for CHWs.

Lastly, the disease-specific nature and small scale of the trainings in our review revealed that the evidence base focuses largely on relatively fragmented approaches to train CHWs. Much focus, in the literature, has been given to what training CHWs can do for communities, and there remains a need to explore what being a trained CHW does for the individual.

### Implications for policy, practice and future research

Well-trained, competent, and well-supported Community Health Workers are an integral component of health workforce teams best placed to achieve Universal Health Coverage. Countries of all income levels will require substantial investment and intentional planning to adequately transition to competency-based education [15].

Broadly speaking, this review illustrates that CHWs can be successfully trained in a broad range of competencies, including for complex tasks like research. Exploring how some of the smaller scale interventions such as those found in our review could be replicated in more large-scale CHW programs could further improve understanding.

The need to tailor trainings across factors like culture, language, and education level was well-documented in our review, with ample creative approaches available to inform curricula design. We also saw evidence for the value of co-design to strengthen curricula. CHWs bring unique expertise as members of the communities they serve; this echoes other calls to invite individuals with lived experience to inform global health curricula [145]. Trainings tailored to the realities of CHW programs are important and can help illuminate for program planners what activities are truly reasonable to expect at various levels of remuneration, supervision, and professionalization, underlining the value of systematic support for CHWs.

Although care for non-communicable diseases (NCDs) is increasingly part of CHWs’ responsibilities globally, our literature identified few articles on competency based-trainings for NCDs in low- and middle-income countries. This suggests the need for work on effective strategies to train CHWs to address the “triple burden” of disease [146] at the intersection of infectious disease, chronic conditions, and broader structural determinants. It also highlights the continued need for research strategies beyond short-term, disease-specific training approaches [147].

We suggest that researchers publishing about trainings provide details on the context of the CHW program. Some key context to report would include program management, the educational requirements of trainees, career progression for trainees, and remuneration for training. More evidence on what educational approaches work in various contexts is needed.

There is a clear need for further published research on effective competency-based education in large-scale CHW programs in low- and middle-income countries with an emphasis on national CHW programs; these programs employ millions of workers and are critical in care provision globally. Yet literature on training programs for these programs is sparse. In particular, there is evidence needed on learning and course outcomes of the current training programs employed in these large-scale programs. Building this evidence base can be a key way of supporting broad-based Primary Health Care.

## Supplementary Information

Supplementary Material 1: Annex 1. PRISMA Scoping Review.

Supplementary Material 2: Annex 2. Detailed Search Strategy.

Supplementary Material 3: Annex 3. Exclusion Criteria for Abstract and Full-Text Screening.

Supplementary Material 4: Annex 4. Data Charting Columns.

Supplementary Material 5: Annex 5. Included Articles Title, Author, Country, Income Level and Region.

## Data Availability

All data generated or analyzed during this study are included in this published article and its supplementary information files.

## Abbreviations

CHW: Community Health Worker
NGO: Non-governmental organization
WHO: World Health Organization
PRISMA-ScrR: Preferred Reporting Items for Systematic Reviews and Meta-Analyses Extension for Scoping Reviews
ISCO: International Standard Classification of Occupations
AMR: Region of the Americas
EUR: European Region
EMR: Eastern Mediterranean Region
SEAR: South-East Asian Region
AFR: African Region
LMIC: Lower-Middle Income Country
LIC: Low Income Country
UMIC: Upper Middle-Income Country
UIC: Upper Income Country
NCD: Non-communicable disease
RNMCH: Reproductive, Neonatal, Maternal and Child Health
WASH: Water, Sanitation and Hygiene
LHW: Lady Health Worker
AC-E: Certified Asthma Educator
EFAR: Emergency first aid responder
