# Supplementary material for: Competency-based education and training for Community Health Workers: a scoping review
Source: BMC Health Serv Res. 2025 Feb 17;25:263. doi: 10.1186/s12913-025-12217-7 (PMC11834664; doi:10.1186/s12913-025-12217-7)
Supplement: Supplementary file 2 — Supplementary Material 2: Annex 2. Detailed Search Strategy. [file 12913_2025_12217_MOESM2_ESM.docx]

Annex 2

Search Strategy by Database

Color-coding:

Bright blue: Community Heath Worker MESH terms, varies by database

Light blue: Community Health Worker cadre titles, same across databases

Bright green: Education MESH terms, varies by database

Light green: Education terms, same across databases

Dull Red: Exclusion and parameters

EMBASE Search Strategy:

('health auxiliary'/exp

OR

(‘Accompagnateur’ OR ‘Accompanier*’ OR ‘Accredited Social Health Activist*’ OR ‘Acompañante*’ OR ‘Activista*’ OR ‘Adherence supporter*’ OR ‘Agente comunitario de salud’ OR ‘Agente comunitário de saúde’ OR ‘Agente polivalente elementare’ OR ‘agente* communitario* de saude’ OR ‘agente* comunitario* de salud’ OR ‘agente* comunitario* de saude’ OR ‘Agente* polivalente* elementare*’ OR ‘agentes de saúde’ OR ‘angan*wadi*’ OR ‘anganawadi*’ OR ‘Anganwadi’ OR ‘Animator’ OR ‘Animatrice’ OR ‘animatrice*’ OR ‘ASHA*’ OR ‘auxiliary health worker*’ OR ‘Auxiliary midwife’ OR ‘Auxiliary Nurse’ OR ‘auxiliary nurse midwi*’ OR ‘barangay health worker*’ OR ‘barefoot doctor*’ OR ‘Basic Health worker*’ OR ‘behvarz*’ OR ‘binome*’ OR ‘Bridge-to-Health Team’ OR ‘Brigadista*’ OR ‘Care Group Volunte*’ OR ‘close-to-community’ OR ‘Colaborad*’ OR ‘Community Case Management worker*’ OR ‘Community drug distribut*’ OR ‘community educator*’ OR ‘community health adviso*’ OR ‘Community health agent*’ OR ‘community health aide*’ OR ‘community health assistant*’ OR ‘Community Health Care Provid*’ OR ‘community health distribut*’ OR ‘community health educator*’ OR ‘Community Health Extension worker*’ OR ‘Community Health Offic*’ OR ‘Community health promotor*’ OR ‘Community health representativ*’ OR ‘community health surveyor*’ OR ‘Community health volunte*’ OR ‘community health worker*’ OR ‘Community HealthCare Provid*’ OR ‘community IMCI’ OR ‘community medical assistant*’ OR ‘community midwi*’ OR ‘community mobiliser*’ OR ‘community mobilizer*’ OR ‘community nutrition worker*’ OR ‘Community Practition*’ OR ‘Community resource person’ OR ‘community skilled birth attendant*’ OR ‘Community Surveillance Volunte*’ OR ‘community volunte*’ OR ‘community#based agent*’ OR ‘community#based mobili*’ OR ‘community#based practition*’ OR ‘community#based worker*’ OR ‘extension worker*’ OR ‘Family Health worker*’ OR ‘Family Planning Agent*’ OR ‘Family Welfare Assist*’ OR ‘Female Community Health Volunte*’ OR ‘Female multipurpose health worker*’ OR ‘frontline health worker*’ OR ‘Health Agent*’ OR ‘Health Assistant*’ OR ‘health extension volunteer*’ OR ‘Health Extension worker*’ OR ‘health promotor*’ OR ‘Health Surveillance Assistant*’ OR ‘Kader’ OR ‘lady health worker*’ OR ‘lay counselor*’ OR ‘lay health worker*’ OR ‘Lead Mother’ OR ‘Malaria Agent*’ OR ‘Maternal and Child Health worker*’ OR ‘mitanin*’ OR ‘Mobile Clinic Team*’ OR ‘mobile health worker*’ OR ‘Monitora’ OR ‘Mother coordinator’ OR ‘multipurpose health worker*’ OR ‘Multipurpose worker*’ OR ‘Nutrition Agent*’ OR ‘Nutrition Counselor*’ OR ‘Nutrition worker*’ OR ‘Outreach educator*’ OR ‘outreach worker*’ OR ‘Peer Educator*’ OR ‘polyvalent health worker*’ OR ‘Promotora*’ OR ‘promotora* de salud’ OR ‘promotore*’ OR ‘public health worker*’ OR ‘raedat*’ OR ‘rural health auxiliar*’ OR ‘Sakhi*’ OR ‘Sevika’ OR ‘Shastho karmis’ OR ‘Shastho shebika’ OR ‘Shasthya Shebika’ OR ‘shebika*’ OR ‘socorrista*’ OR ‘traditional birth attendant*’ OR ‘Village drug*kit manager’ OR ‘village health agent*’ OR ‘village health guide*’ OR ‘Village health helper’ OR ‘village health volunteer*’ OR ‘village health worker*’ OR ‘village midwi*’ OR ‘voluntario’ OR ‘voluntary health worker*’ OR ‘volunteer health worker*’):ti,ab)

AND

(('continuing education'/exp OR ‘curriculum'/exp OR 'professional development'/exp OR 'vocational guidance'/exp OR 'accreditation'/exp OR 'in service training'/exp OR 'mentoring'/exp OR 'capacity building'/exp OR 'interprofessional education'/exp)

OR

(‘accreditation*’ OR ‘capacity-building’ OR ‘career progression’ OR ‘competency#based certification ’ OR ‘competency#based curric*’ OR ‘competency#based education’ OR ‘continuing#education’ OR ‘curric*’ OR ‘in#service course’ OR ‘in#service train*’ OR ‘inter*professional education’ OR ‘life#long education’ OR ‘life#long training’ OR ‘lifelong learning’ OR ‘mentoring’ OR ‘mentorship’ OR ‘midwifery#education’ OR ‘modular#training’ OR ‘nurs* training’ OR ‘paramedical education’ OR ‘paramedical training’ OR ‘performance measure*’ OR ‘pre#service course’ OR ‘pre#service train*’ OR ‘professional development’ OR ‘professional education’ OR ‘refresher#training’ OR ‘supportive#supervis*’ OR ‘teacher#education’ OR ‘teacher#training’ OR ‘train#the#trainer*’ OR ‘training program*’ OR ‘training#curric*’ OR ‘training#the#trainer*’ OR ‘vocational education’ OR ‘vocational school’ OR ‘vocational training’ OR ‘workforce readi*’):ti,ab)

AND

[2010-2023]/PY AND english:la AND('article'/it OR 'article in press'/it OR 'preprint'/it OR 'review'/it)

OVID MEDLINE Search Strategy:

Ovid MEDLINE(R) ALL <1946 to March 23, 2023>

1        (exp Community Health Workers/ or (((Accompagnateur or Accompanier* or Accredited Social Health Activist* or Acompanante* or Activista* or Adherence supporter* or Agente comunitario de salud or Agente comunitario de saude or Agente polivalente elementare or agente* communitario* de saude or agente* comunitario* de salud or agente* comunitario* de saude or Agente* polivalente* elementare* or agentes de saude or anganwadi* or anganawadi* or Anganwadi or Animator or Animatrice or animatrice* or ASHA* or auxiliary health worker* or Auxiliary midwife or Auxiliary Nurse or auxiliary nurse midwi* or barangay health worker* or barefoot doctor* or Basic Health worker* or behvarz* or binome* or Bridge-to-Health Team or Brigadista* or Care Group Volunte* or close-to-community or Colaborad* or Community Case Management worker* or Community drug distribut* or community educator* or community health adviso* or Community health agent* or community health aide* or community health assistant* or Community Health Care Provid* or community health distribut* or community health educator* or Community Health Extension worker* or Community Health Offic* or Community health promotor* or Community health representativ* or community health surveyor* or Community health volunte* or community health worker* or Community HealthCare Provid* or community IMCI or community medical assistant* or community midwi* or community mobiliser* or community mobilizer* or community nutrition worker* or Community Practition* or Community resource person or community skilled birth attendant* or Community Surveillance Volunte* or community volunte* or community based agent* or community based mobili* or community based practition* or community based worker* or community-based agent* or community-based mobili* or community-based practition* or community-based worker* or extension worker* or Family Health worker* or Family Planning Agent* or Family Welfare Assist* or Female Community Health Volunte* or Female multipurpose health worker* or frontline health worker* or Health Agent* or Health Assistant* or health extension volunteer* or Health Extension worker* or health promotor* or Health Surveillance Assistant* or Kader or lady health worker* or lay counselor* or lay health worker* or Lead Mother or Malaria Agent* or Maternal) and Child Health worker*) or mitanin* or Mobile Clinic Team* or mobile health worker* or Monitora or Mother coordinator or multipurpose health worker* or Multipurpose worker* or Nutrition Agent* or Nutrition Counselor* or Nutrition worker* or Outreach educator* or outreach worker* or Peer Educator* or polyvalent health worker* or Promotora* or promotora* de salud or promotore* or public health worker* or raedat* or rural health auxiliar* or Sakhi* or Sevika or Shastho karmis or Shastho shebika or Shasthya Shebika or shebika* or socorrista* or traditional birth attendant* or Village drug*kit manager or village health agent* or village health guide* or Village health helper or village health volunteer* or village health worker* or village midwi* or voluntario or voluntary health worker* or volunteer health worker*).ti,ab,kw.) and (Competency-Based Education/ or Vocational Education/ or Education, Professional/ or Education, Medical/ or Education, Nursing/ or Education, Public Health Professional/ or Teacher Training/ or Inservice Training/ or Staff Development/ or Curriculum/ or Mentoring/or (accreditation* or capacity-building or career progression or Competency based certification or competency based curric* or competency based education or continuing education or curric* or in service course or in service train* or Competency-based certification or competency-based curric* or competency-based education or continuing-education or curric* or in-service course or in-service train* or inter*professional education or lifelong education or lifelong training or life-long education or life-long training or lifelong learning or life-long learning or mentoring or mentorship or midwifery education or modular-training or nurs* training or paramedical education or paramedical training or performance measure* or pre-service course or pre-service train* preservice course or preservice train* or professional development or professional education or refresher-training or supportive-supervis* or teacher-education or teacher-training or train-the-trainer* or refresher training or supportive supervis* or teacher education or teacher training or train the trainer* or training program* or training curric* or training the trainer* or vocational education or vocational school or vocational training or workforce readi*).ti,ab,kw.) and english.lg. and (randomized controlled trial or "review" or "systematic review" or technical report or validation study or case reports or clinical study or clinical trial or comparative study or evaluation studies or evaluation study or government publication or journal article or meta analysis or multicenter study or observational study or practice guideline or preprint).pt.

limit 1 to yr="2010 – 2023”

Web of Science Search Strategy:

AB=(“Accompagnateur” or “Accompanier*” or “Accredited Social Health Activist*” or “Acompanante*” or “Activista*” or “Adherence supporter*” or “Agente comunitario de salud” or “Agente comunitario de saude” or “Agente polivalente elementare” or “agente* communitario* de saude” or “agente* comunitario* de salud” or “agente* comunitario* de saude” or “Agente* polivalente* elementare*” or “agentes de saude” or “anganwadi*” or “anganawadi*” or “Anganwadi” or “Animator” or “Animatrice” or “animatrice*” or “ASHA*” or “auxiliary health worker*” or “Auxiliary midwife” or “Auxiliary Nurse” or “auxiliary nurse midwi*” or “barangay health worker*” or “barefoot doctor*” or “Basic Health worker*” or “behvarz*” or “binome*” or “Bridge-to-Health Team” or “Brigadista*” or “Care Group Volunte*” or “close-to-community” or “Colaborad*” or “Community Case Management worker*” or “Community drug distribut*” or “community educator*” or “community health adviso*” or “Community health agent*” or “community health aide*” or “community health assistant*” or “Community Health Care Provid*” or “community health distribut*” or “community health educator*” or “Community Health Extension worker*” or “Community Health Offic*” or “Community health promotor*” or “Community health representativ*” or “community health surveyor*” or “Community health volunte*” or “community health worker*” or “Community HealthCare Provid*” or “community IMCI” or “community medical assistant*” or “community midwi*” or “community mobiliser*” or “community mobilizer*” or “community nutrition worker*” or “Community Practition*” or “Community resource person” or “community skilled birth attendant*” or “Community Surveillance Volunte*” or “community volunte*” or “community based agent*” or “community based mobili*” or “community based practition*” or “community based worker*” or “community-based agent*” or “community-based mobili*” or “community-based practition*” or “community-based worker*” or “extension worker*” or “Family Health worker*” or “Family Planning Agent*” or “Family Welfare Assist*” or “Female Community Health Volunte*” or “Female multipurpose health worker*” or “frontline health worker*” or “Health Agent*” or “Health Assistant*” or “health extension volunteer*” or “Health Extension worker*” or “health promotor*” or “Health Surveillance Assistant*” or “Kader” or “lady health worker*” or “lay counselor*” or “lay health worker*” or “Lead Mother” or “Malaria Agent*” or “Maternal) and Child Health worker*)” or “mitanin*” or “Mobile Clinic Team*” or “mobile health worker*” or “Monitora” or “Mother coordinator” or “multipurpose health worker*” or “Multipurpose worker*” or “Nutrition Agent*” or “Nutrition Counselor*” or “Nutrition worker*” or “Outreach educator*” or “outreach worker*” or “Peer Educator*” or “polyvalent health worker*” or “Promotora*” or “promotora* de salud” or “promotore*” or “public health worker*” or “raedat*” or “rural health auxiliar*” or “Sakhi*” or “Sevika” or “Shastho karmis” or “Shastho shebika” or “Shasthya Shebika” or “shebika*” or “socorrista*” or “traditional birth attendant*” or “Village drug*kit manager” or “village health agent*” or “village health guide*” or “Village health helper” or “village health volunteer*” or “village health worker*” or “village midwi*” or “voluntario” or “voluntary health worker*” or “volunteer health worker*”)

AND

AB=(“accreditation*” OR “capacity-building” OR “career progression” or “Competency based certification” or “competency based curric*” or “competency based education” or “continuing education” or “curric*” or “in service course” or “in service train*” or “Competency-based certification” or “competency-based curric*” or “competency-based education” or “continuing-education” or “curric*” or “in-service course” or “in-service train*” or “inter*professional education” or “lifelong education” or “lifelong training” or “life-long education” or “life-long training” or “lifelong learning” or “life-long learning” or “mentoring” or “mentorship” or “midwifery education” or “modular-training” or “nurs* training” or “paramedical education” or “paramedical training” or “performance measure*” or “pre-service course” or “pre-service training” or “preservice course” or “preservice training” or “professional development” or “professional education” or “refresher-training” or “supportive-supervision” or “teacher-education” or “teacher-training” or “train-the-trainer” or “refresher training” or “supportive supervision” or “teacher education” or “teacher training” or “train the trainer” or “training program” or “training curriculum” or “training the trainer*” or “vocational education” or “vocational school” or “vocational training” or “workforce readi*”)

AND

PY=(2010-2023) AND LA=(english)

Document types: article, review article, early access

Citation Topics Meso : healthcare policy or education or health literacy and telemedicine

CINHAL Search Strategy:

( (((MH "Community Health Workers") ) OR (TX (Accompagnateur or Accompanier* or Accredited Social Health Activist* or Acompanante* or Activista* or Adherence supporter* or Agente comunitario de salud or Agente comunitario de saude or Agente polivalente elementare or agente* communitario* de saude or agente* comunitario* de salud or agente* comunitario* de saude or Agente* polivalente* elementare* or agentes de saude or anganwadi* or anganawadi* or Anganwadi or Animator or Animatrice or animatrice* or ASHA* or auxiliary health worker* or Auxiliary midwife or Auxiliary Nurse or auxiliary nurse midwi* or barangay health worker* or barefoot doctor* or Basic Health worker* or behvarz* or binome* or Bridge-to-Health Team or Brigadista* or Care Group Volunte* or close-to-community or Colaborad* or Community Case Management worker* or Community drug distribut* or community educator* or community health adviso* or Community health agent* or community health aide* or community health assistant* or Community Health Care Provid* or community health distribut* or community health educator* or Community Health Extension worker* or Community Health Offic* or Community health promotor* or Community health representativ* or community health surveyor* or Community health volunte* or community health worker* or Community HealthCare Provid* or community IMCI or community medical assistant* or community midwi* or community mobiliser* or community mobilizer* or community nutrition worker* or Community Practition* or Community resource person or community skilled birth attendant* or Community Surveillance Volunte* or community volunte* or community based agent* or community based mobili* or community based practition* or community based worker* or community-based agent* or community-based mobili* or community-based practition* or community-based worker* or extension worker* or Family Health worker* or Family Planning Agent* or Family Welfare Assist* or Female Community Health Volunte* or Female multipurpose health worker* or frontline health worker* or Health Agent* or Health Assistant* or health extension volunteer* or Health Extension worker* or health promotor* or Health Surveillance Assistant* or Kader or lady health worker* or lay counselor* or lay health worker* or Lead Mother or Malaria Agent* or Maternal) and Child Health worker*) or mitanin* or Mobile Clinic Team* or mobile health worker* or Monitora or Mother coordinator or multipurpose health worker* or Multipurpose worker* or Nutrition Agent* or Nutrition Counselor* or Nutrition worker* or Outreach educator* or outreach worker* or Peer Educator* or polyvalent health worker* or Promotora* or promotora* de salud or promotore* or public health worker* or raedat* or rural health auxiliar* or Sakhi* or Sevika or Shastho karmis or Shastho shebika or Shasthya Shebika or shebika* or socorrista* or traditional birth attendant* or Village drug*kit manager or village health agent* or village health guide* or Village health helper or village health volunteer* or village health worker* or village midwi* or voluntario or voluntary health worker* or volunteer health worker*))) AND (((MH "Education, Competency-Based") OR (MH "Education, Midwifery") OR (MH "Education, Continuing+") OR (MH "Curriculum+") OR (MH "Professional Knowledge+")) OR ((MH "Credentialing Examinations") OR (MH "Professional Competence") OR (((accreditation* or capacity or career progression or Competency based certification or competency based curric* or competency based education or continuing education or curric* or in service course or in service train* or Competency-based certification or competency-based curric* or competency-based education or continuing-education or curric* or in-service course or in-service train* or inter*professional education or lifelong education or lifelong training or life-long education or life-long training or lifelong learning or life-long learning or mentoring or mentorship or midwifery education or modular-training or nurs* training or paramedical education or paramedical training or performance measure* or pre-service course or pre-service train* preservice course or preservice train* or professional development or professional education or refresher-training or supportive-supervis* or teacher-education or teacher-training or train-the-trainer* or refresher training or supportive supervis* or teacher education or teacher training or train the trainer* or training program* or training curric* or training the trainer* or vocational education or vocational school or vocational training or workforce readi*)) ) ))) AND PY 2010- 2022 AND LA English

Limit to Academic journals
