# Supplementary material for: Competency-based education and training for Community Health Workers: a scoping review
Source: BMC Health Serv Res. 2025 Feb 17;25:263. doi: 10.1186/s12913-025-12217-7 (PMC11834664; doi:10.1186/s12913-025-12217-7)
Supplement: Supplementary file 3 — Supplementary Material 3: Annex 3. Exclusion Criteria for Abstract and Full-Text Screening. [file 12913_2025_12217_MOESM3_ESM.docx]

Annex 3

Exclusion Criteria for Abstract and Full-Text Screening

| Duplicate | Make decision on 1 of the articles, EXCL the rest with reason - "duplicate". Label Duplicate SET, i.e. each/all article(s) of which there are more than 1 copy. |
| --- | --- |
| Excluded language | Included results must be in English |
| Excluded publication type | Conference abstract, Commentary/Opinion/Viewpoint, Book, Book chapter, Editorial, Letter to the Editor, Protocol/Methods articles |
| Not CHW | Article does not focus on Community Health Workers, as per our agreed definition. |
| Not Education or Training | Article focuses on CHWs but focus is on patient outcomes, stakeholder (e.g. policymaker, program manager) perspectives on aspects other than CHW training and competency-building. Exclude training/skill evaluation articles that do not include details of the training in methods section. |
| Not Competency-Based* | The education described in the article does not include at least 2 of the following dimensions: knowledge, skills, attitudes, beliefs. |

*Added during Full-Text Screen
