# Supplementary material for: Competency-based education and training for Community Health Workers: a scoping review
Source: BMC Health Serv Res. 2025 Feb 17;25:263. doi: 10.1186/s12913-025-12217-7 (PMC11834664; doi:10.1186/s12913-025-12217-7)
Supplement: Supplementary file 4 — Supplementary Material 4: Annex 4. Data Charting Columns. [file 12913_2025_12217_MOESM4_ESM.docx]

Annex 4

Data Charting Columns

| Category | Columns |
| --- | --- |
| Article Information | Database ID; source; title; authors; study design; key findings; other details |
| Location | Country; World Bank income designation (2021); name of CHW program; other location details (region was later extrapolated from country) |
| Who? | Trainee Population Features:  Number trained; educational or other requirements of new trainees; workforce renumeration type |
| How? | Training Methods: Program management, pre-service/in-service, length; sections; quality assurance; evaluation; post-training support; trainee assessment; practicum/clinical experience. |
| What? | Training Content: Roles, health services; practice areas; other domains; practice area/domain descriptions; competency descriptions |
| Towards What Ends? | Formal credential; remuneration for training; career progression pathway |
